# Liver fatty acid composition in mice with or without nonalcoholic fatty liver disease

**DOI:** 10.1186/1476-511X-10-234

**Published:** 2011-12-14

**Authors:** Xin Wang, Yuzhen Cao, Yunwei Fu, Guifang Guo, Xiuying Zhang

**Affiliations:** 1 College of Animal Sciences and Veterinary Medicine, Heilongjiang Bayi Agricultural University, Daqing, China 163319; 2 College of Veterinary Medicine, Northeast Agricultural University, Harbin, China 150030; 3 China Institute of Veterinary Drugs Control, Beijing, China 100080

**Keywords:** Fatty acid, Nonalcoholic fatty liver disease, Mouse, High-fat feed, Carbon tetrachloride

## Abstract

**Background:**

Nonalcoholic fatty liver disease (NAFLD) is one of the most frequent causes of abnormal liver function. Because fatty acids can damage biological membranes, fatty acid accumulation in the liver may be partially responsible for the functional and morphological changes that are observed in nonalcoholic liver disease. The aim of this study was to use gas chromatography-mass spectrometry to evaluate the fatty acid composition of an experimental mouse model of NAFLD induced by high-fat feed and CCl_4 _and to assess the association between liver fatty acid accumulation and NAFLD. C57BL/6J mice were given high-fat feed for six consecutive weeks to develop experimental NAFLD. Meanwhile, these mice were given subcutaneous injections of a 40% CCl_4_-vegetable oil mixture twice per week.

**Results:**

A pathological examination found that NAFLD had developed in the C57BL/6J mice. High-fat feed and CCl_4 _led to significant increases in C14:0, C16:0, C18:0 and C20:3 (P < 0.01), and decreases in C15:0, C18:1, C18:2 and C18:3 (P < 0.01) in the mouse liver. The treatment also led to an increase in SFA and decreases in other fatty acids (UFA, PUFA and MUFA). An increase in the ratio of product/precursor n-6 (C20:4/C18:2) and n-3 ([C20:5+C22:6]/C18:3) and a decrease in the ratio of n-6/n-3 (C20:4/[C20:5+C22:6]) were also observed.

**Conclusion:**

These data are consistent with the hypothesis that fatty acids are deranged in mice with non-alcoholic fatty liver injury induced by high-fat feed and CCl_4_, which may be involved in its pathogenesis and/or progression via an unclear mechanism.

## Background

Nonalcoholic fatty liver disease (NAFLD) encompasses a spectrum of conditions that are histologically characterized by hepatic steatosis in individuals without significant alcohol consumption and with no viral, congenital, or autoimmune liver disease markers [1]. It is associated with insulin resistance and metabolic syndrome [2,3]. Despite the many possible etiologies of NAFLD [4,5], these results reflect the accumulation of lipids within the hepatocyte cytoplasm.

High-fat feed ingestion and hepatic toxins (such as CCl_4_) may lead to fatty acid accumulation and hepatic damage. Hepatic lipid accumulation in hepatocytes (hepatic steatosis) is the hallmark of NAFLD and an important factor that can induce insulin resistance, lipid peroxidation, changes in energy metabolism, hepatic cell damage and inflammation. Fatty acid are the simplest lipids. They are the basic components of more complex lipids (including triglycerides, phospholipids and sphingolipids) and an important metabolic fuel. The compositions of the lipids that accumulate in livers of subjects with NAFLD are not well characterized. Most of the published literature has focused on triglycerides accumulation as the key defect in NAFLD [6,7]. However, it is unknown whether there are substantial changes in other lipid classes, such as fatty acid. Although an increase in the n-6/n-3 fatty acid ratio in the total lipids has been observed in NAFLD [8,9], the composition of fatty acid in the hepatic lipids has not been extensively characterized.

The pathogenic mechanism involved in the development of fatty liver is unclear. In alcoholic patients with asymptomatic fatty liver and in morbidly obese patients, free fatty acid accumulation was observed in liver extracts [10,11]. Non-alcoholic steatohepatitis patients had significantly higher concentrations of total and free fatty acid in their plasma, compared with healthy individuals [12]. Changes in the fatty lipid composition may be implicated in the pathogenesis of NAFLD. As yet, there is little information available concerning the hepatic lipid fatty acid composition in NAFLD [13]. The aim of this study was to compare the liver fatty acid profiles and detailed compositions of healthy mice vs. mice with an experimental model of NAFLD induced by high-fat feed and CCl_4_.

## Results and discussion

### Histological profile

**Figure 1:**
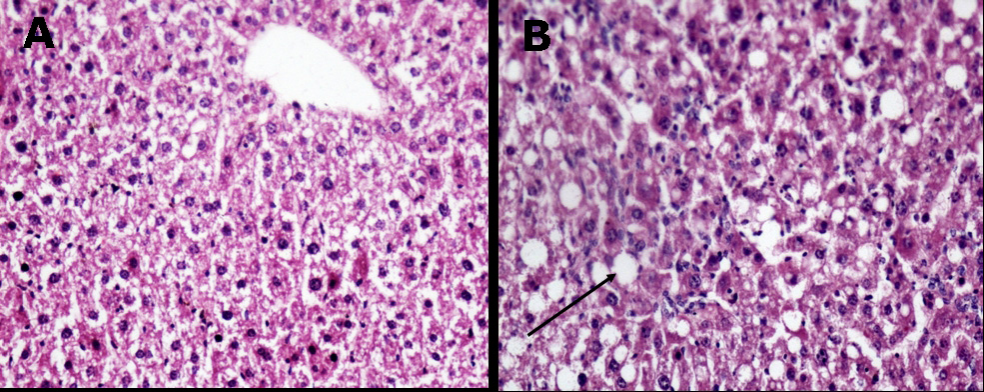
**Histological analysis of liver sections from mice that were fed a high-fat feed and control mice**. Fresh sections were stained with H&E to demonstrate lipid accumulation. (A) Control mouse liver. (B) Experimental mouse liver with severe hepatosteatosis consisting of mixed microvesicular and macrovesicular fat accumulation.

All of the sections in the experimental group exhibited diffuse hepatic steatosis **(**Figure 1**) **under a light microscope, whereas no fatty liver was observed in the control group The relative sizes of the hepatic cell nuclei were uneven. Hepatic steatosis (mostly microvesicular and macrovesicular mixed steatosis) was most obvious around the portal area and was accompanied by liver cell necrosis and inflammatory cell infiltration. The lobular and portal areas exhibited considerably more inflammatory cell infiltration in the experimental group than in the control group The total histological scores of the livers in the model-group mice reached grade 2 or 3. In contrast to the control mice, a histological analysis of the livers from the mice treated with a high-fat feed and a hepatotoxin (CCl_4_) confirmed marked fat accumulation and revealed extensive inflammatory cell infiltration, indicating that diffuse hepatic steatosis with moderate inflammation (NAFLD) had developed.

### Serological profiles

Twenty mice in each group were studied (Table 1). The mice with NAFLD had significantly higher TC, HDL, LDL, ALP, AST and ALT levels than did the mice in the control group (P < 0.01), whereas the ALB level was higher in mice with NAFLD than it was in control mice, but this difference did not reach statistical significance. However, the TG, TP and glucose levels were significantly lower in the mice with NAFLD than in the control mice (P < 0.05 or P < 0.01).

**Table 1:** Serum characteristics of mice with or without NAFLD

Parameters	Controls	Mice with NAFLD
TG (mmol/L)	0.74 ± 0.12	0.61 ± 0.11**
TC (mmol/L)	2.97 ± 0.35	4.65 ± 0.81**
HDL (mmol/L)	2.16 ± 0.43	2.87 ± 0.56**
LDL (mmol/L)	0.41 ± 0.09	0.83 ± 0.17**
TP (g/L)	82.69 ± 9.31	71.18 ± 9.45**
ALB (g/L)	39.67 ± 4.08	42.06 ± 2.33
GLU (mmol/L)	1.34 ± 0.32	0.94 ± 0.37*
ALT (IU/L)	64.71 ± 8.56	198.62 ± 12.36**
AST (IU/L)	56.32 ± 9.83	143.27 ± 17.38**
ALP (IU/L)	114.06 ± 13.62	149.83 ± 19.75**

The data are expressed as the mean ± SD (n = 20 per treatment group). *P < 0.05, ** *P *< 0.01 vs. control. ALT, alanine aminotransferase; AST, aspartate aminotransferase; ALP, alkaline phosphatase; TG, triglycerides; TP, total protein; HDL, high-density lipoprotein; LDL, low-density lipoprotein; TC, total cholesterol; ALB, albumin; NAFLD, nonalcoholic fatty liver disease.

This study have showed that high-fat feed and CCl_4 _have pronounced effects on hepatic steatosis, decrease the plasma TG level, and induce hepatocyte necrosis and inflammatory cell infiltration. Serological and pathological examinations confirmed that non-alcoholic fatty liver injury developed in C57BL/6J mice in our study. The blood levels of transaminase (AST, ALT and ALP) were significantly elevated in the model mice, indicating liver dysfunction and confirming the widely held belief that serum transaminase is a sensitive indicator of liver injury. The concentrations of serum TG and TC are known to vary, depending on pathological factors [14]. Our results revealed significantly higher TC levels (P < 0.01) and significantly lower TG levels in the plasma of the model mice than in the plasma of control mice. Decreased circulating TG in the model mice indicated impaired hepatic export of TG due to the high-fat feed and CCl_4_. This result does not support the evidence reported by Das and De Almeida [12,15].

### Hepatic fatty acid composition

The hepatic fatty acid compositions of the mice with or without NAFLD are given in Table 2. The concentrations of C14:0, C16:0, C18:0 and C20:3 were significantly increased in the experimental group (P < 0.01), compared with the control group, whereas the concentrations of C18:1, C18:2 and C18:3 were significantly decreased (P < 0.01). The SFA concentration was higher in experimental group than in the control group (P < 0.01); this difference was largely due to significant increases in C14:0, C16:0 and C18:0. While the polyunsaturated fatty acid (PUFA) and monounsaturated fatty acid (MUFA) concentrations were lower in the mice in the experimental group than in the mice in the control group (P < 0.01), this difference were mainly due to the significant decreases (P < 0.01) in the C18:2, C18:3 and C20:4 PUFA and the C18:1 MUFA.

**Table 2:** The fatty acid content and ratios in the mice liver

Fatty acid	Control group	Experimental group
C14:0 (μg/mg)	0.47 ± 0.13	2.39 ± 0.66**
C15:0 (μg/mg)	0.34 ± 0.12	0.18 ± 0.04**
C16:0 (μg/mg)	23.41 ± 7.72	70.08 ± 17.95**
C17:0 (μg/mg)	0.35 ± 0.11	0.41 ± 0.10
C18:0 (μg/mg)	11.07 ± 4.07	17.33 ± 5.15**
C18:1 n-9 (μg/mg)	93.87 ± 19.62	56.69 ± 19.08**
C18:2 n-6 (μg/mg)	84.96 ± 27.43	42.12 ± 22.11**
C18:3 n-3 (μg/mg)	2.76 ± 1.43	1.07 ± 0.66**
C20:1 n-9 (μg/mg)	0.94 ± 0.34	0.97 ± 0.38
C20:3 n-6 (μg/mg)	1.19 ± 0.44	2.10 ± 0.78**
C20:4 n-6 (μg/mg)	14.89 ± 5.18	11.10 ± 4.37
C20:5 n-3 (μg/mg)	0.64 ± 0.21	1.06 ± 0.57
C22:6 n-3 (μg/mg)	7.36 ± 4.76	6.57 ± 3.95
TFA (μg/mg)	242.24 ± 14.44	212.79 ± 11.95**
SFA (μg/mg)	35.64 ± 6.15	91.11 ± 6.92**
UFA (μg/mg)	206.60 ± 14.62	121.68 ± 12.07**
MUFA (μg/mg)	94.81 ± 10.53	57.66 ± 11.46**
PUFA (μg/mg)	111.80 ± 10.51	64.02 ± 8.38**
SFA/TFA ratio (%)	14.73 ± 2.44	42.89 ± 3.26**
UFA/TFA ratio (%)	85.27 ± 2.44	57.11 ± 3.43**
PUFA/SFA ratio (%)	323.23 ± 66.64	70.03 ± 11.05**
MUFA/SFA ratio (%)	273.63 ± 59.59	63.91 ± 14.62**
n-6/n-6 ratio (%)	18.04 ± 5.04	28.15 ± 8.90**
n-3/n-3 ratio (%)	286.69 ± 67.02	740.80 ± 58.75**
n-6/n-3 ratio (%)	188.29 ± 15.51	143.33 ± 13.66**

All of the values are expressed as mean ± standard deviation (SD) (n = 20 per treatment group). TFA, total fatty acid; SFA, saturated fatty acid; UFA, unsaturated fatty acid; PUFA, polyunsaturated fatty acid; MUFA, monounsaturated fatty acid; n-6/n-6 ratio, C20:4/C18:2; n-3/n-3 ratio, (C20:5+ C22:6)/C18:3; n-6/n-3 ratio, C20:4/(C20:5+C22:6). **P < 0.01 vs. the control group.

The ratio of product/precursor n-6 (C20:4/C18:2) and n-3 ([C20:5 + C22:6]/C18:3) in the mice in the experimental group was higher than in the control group (P < 0.01), whereas the ratio of n-6/n-3 (20:4/[C20:5 + C22:6]) in the mice in the experimental group was lower than in the control group. In addition, the SFA/TFA ratio was increased, and the UFA/TFA, PUFA/TFA, PUFA/SFA and MUFA/SFA ratios were decreased **(**Table 2**)**.

High-fat feed has become popular in recent years as a way to induce model of hepatic steatosis and steatohepatitis [16]. The accompaniment of a high-fat feed and CCL_4_-mediated accumulation of fatty acid by hepatocyte necrosis and inflammatory cell infiltration was unusual because most other models of fatty acid accumulation have been induced by high-fat feed [17]. High-fat feed and CCl_4 _would be expected to produce hepatic fatty acid accumulation and liver injury because the ingestion of a large amount of fat and hepatic toxins (such as CCl_4_) may trigger fatty acid accumulation. Mice with NAFLD are a useful model to examine potential pathogenic factors for non-alcoholic liver damage.

Altered lipid homeostasis in the liver is the pathophysiological hallmark of NAFLD [18]. This study, which was conducted in model mice with histological and serological NAFLD, aimed to investigate the amounts and types of fatty acid that accumulate within the liver in NAFLD and whether their compositions might provide some novel and interesting insights into the pathophysiology of NAFLD. Our findings clearly show that mice with NAFLD induced by a high-fat feed and a hepatotoxin (CCl_4_) exhibit fatty acid accumulation in the liver. Furthermore, relatively similar profiles of SFA, PUFA and MUFA were found in the hepatic fatty acid fractions of the model and control groups. However, hexadecanoic acid (C16:0) and stearic acid (C18:0) were the predominant saturated fatty acid, whereas octadecenoic acid (C18:1) and linoleic acid (C18:2) were the predominant monounsaturated and polyunsaturated fatty acid, respectively. The amounts of octadecenoic acid (C18:1) and linoleic acid (C18:2) in both fractions were significantly lower in the NAFLD group than in the control group, whereas the amounts of C16:0 and C18:0 in the saturated fraction were significantly higher in the NAFLD group than in the control group. The high levels of C16:0 and C18:0 may due to the lard, which is rich in SFA, and they are important factors that can lead to an increase in SFA. In addition, CYP450 can convert CCl_4 _into chloroform radicals (CCl_3_•), which can inhibit Δ-9 desaturase activity in the liver cell membrane and block the conversion of C16:0 and C18:0 into C16:1 and C18:1, respectively [19]. That may also play an active role in the accumulation of C16:0 and C18:0 in the liver. Indeed, liver damage has been ascribed to the direct or indirect toxic effects of SFA accumulation in the liver [20,21]. Barreyro et al showed that excessive SFA (C16:0 and C18:0) accumulation in hepatocytes could induce *Bim *and *FasL *expression, increase stress in the endoplasmic reticulum, and cause hepatocyte injury [22]. Another observation in this study is that increased SFA in liver extracts is accompanied by decreased MUFAs and a decrease in the MUFA/SFA ratio. Saturated fatty acid accumulation at the expense of MUFA has previously been reported in MCD-fed mice, [11] suggesting that MUFA depletion may contribute to liver injury. NAFLD is associated with several factors, such as high saturated fat and cholesterol, which inhibit Δ-6 and Δ-5 desaturase activity [23,24]. We observed fatty acid accumulation in the liver and decreased TG in the plasma, which may be implicated in the pathogenesis of NAFLD.

Thus far, there are no available data regarding the pattern of hepatic fatty acid in NAFLD patients. De Almeida et al. compared the plasma fatty acid of NASH patients and control patients [12]. Their results showed that NASH patients had a significantly higher concentration of free fatty acid, higher total saturated and monounsaturated levels in both lipid fractions, and increased hexadecanoic acid (C16:0) and octadecenoic acid (C18:1) levels, compared with control patients. There was also a progressive increase in MUFA (mainly hexadecanoic and octadecenoic acid) that was associated with the severity of the hepatic lesion [11]. Despite an increase in hexadecanoic acid levels in NAFLD mice, the octadecenoic acid content of the liver was decreased in our study. The roles of these factors are a subject for future studies.

One of the potentially important observations is related to the PUFA changes in the NAFLD model. There was a decrease in the downstream n-6 (arachidonic acid, 20:4n-6) and n-3 (linolenic acid, 18:3 n-3, and docosahexanoic acid, 22:6 n-3) fatty acid in the NAFLD model, which led to a decrease in the total amount of PUFA and the PUFA/TFA and PUFA/SFA ratios. These data suggest that a reduction in these fatty acid may be a compensatory response to hepatotoxicity. This is supported by the possibility that a hepatotoxin (CCl_4_) and NASH also decrease arachidonic acid and docosahexaenoic acid levels [13,25]. Because these fatty acid are precursors for eicosanoids and other bioactive molecules [26], decreased levels of these fatty acid may indicate increased levels of eicosanoids, which play a role in the progression or amelioration of hepatotoxicity. Studies by Capanni et al. have shown that n-3 PUFA significantly decrease fat accumulation in NAFLD mice [27]. Interestingly, linoleic acid has previously been shown to act as an endogenous anti-inflammatory molecule [28], which may indicate that the decreased level of linoleic acid in our high-fat-induced NAFLD model is also associated with hepatotoxicity. Our results reinforce the widely held belief that PUFA may play negative regulatory roles in the pathogenesis of NAFLD [29].

Arachidonic acid (20:4 n-6) is released from membrane PLs by phospholipase A2 and from phosphatidylinositol bisphosphate (through DAG) by phospholipase C. Cyclooxygenase then rapidly converts arachidonic acid into a pro-inflammatory metabolite that accelerates the progression of hepatotoxicity [28,30]. It is possible that the increased utilization of arachidonic acid (20:4 n-6) may also contribute to the observed decrease in arachidonic acid levels in NAFLD. If this is true, phospholipase and cyclooxygenase modulation may provide another mechanism to control the inflammatory pathways of NAFLD.

It must be pointed out that the biological implications of the changes in lipid composition are likely complex and difficult to predict simply on the basis of the fatty acid data. The biological effects of lipids depend on their locations (membrane, cytosolic or nuclear) and amounts [31,32]. They may function as key transcriptional regulators (such as PUFA binding to sterol regulatory element binding protein-1c) or as regulators of enzyme activity (such as PUFA affecting lipid oxidation). Therefore, these hypothesis needs to be confirmed in more focused studies.

The potential limitations of this study are natural NAFLD in the model mice and the small study population size of the test mice. It is possible that the lipid composition of the liver in the model mice may be different from that of NAFLD patients, and the changes observed in NAFLD mice may be even greater than those observed in NAFLD patients. Despite the small study population size, several findings reached statistical significance.

## Conclusions

In summary, this study provides several observations about the alterations in the fatty acid composition in mice with NAFLD induced by high-fat feed and CCl_4_. These observations may be significant for understanding the pathophysiology of this condition. This study demonstrates altered fatty acid compositions in mice with or without NAFLD, an increase in SFA levels and a decrease in UFA (MUFA and PUFA), arachidonic acid, key n-3 fatty acid and the (n-6)/(n-3) fatty acid ratio. All of these observations could play roles in the pathogenesis of NAFLD. They also provide a rationale for the use of n-3 fatty acid supplementation as a treatment for NAFLD.

## Methods

### Drugs and reagents

All of the analytical-grade chemical reagents used in this study were purchased from Tianjin Qianchen Weiye Technology Development Ltd. (Tianjin, China), unless otherwise indicated. The saturated fatty acid (SFA) and unsaturated fatty acid (UFA) standards were purchased from Sigma Chemical Company (St. Louis, MO, USA). All of test kits used in this study were purchased from HuiFeng (S.H.) Medical Science & Technology Co., Ltd. (Shanghai, China), unless otherwise indicated.

### Mouse treatments

Male C57BL/6J mice (9 to 10 weeks old) were purchased from the Animal Center of Heilongjiang Provincial Cancer Hospital (Harbin, China), with the following production license number: SCXK (HEI) 2006-008. The mice were individually housed in our laboratory facility in polycarbonate cages at a controlled temperature (21 to 25°C) and humidity (40 to 70%), with a twelve hour light/dark cycle and free access to sterilized water and a standard pellet diet. After a one-week adaptive period, forty mice were randomly divided into a control group (n = 20), which was fed a standard diet, and a experimental group (n = 20), which was fed a high-fat feed (68.5% normal diet, 15% lard, 1% cholesterol, 0.5% bile and 15% dextrin) for six consecutive weeks and, simultaneously, subcutaneously injected with a 40% CCl_4_-vegetable oil solution (a 0.07 mL/10 g dose in the first week and a 0.04 mL/10 g dose twice per week for the remainder of the study). High-fat feed ingestion for six consecutive weeks has been shown to induce hepatic lipid accumulation in our laboratory (data not shown), and this dose of CCl_4 _has been shown to induce the development of hepatic steatosis [33,34]. Twenty-four hours after the final treatment, the mice were fasted to promote lipid accumulation. The mice were euthanized with CO_2 _gas. At the time of death, blood was collected from the thoracic aorta. The serum was separated from the cellular elements by centrifugation. The livers were removed, rinsed in ice-cold saline, and divided for various assays, as outlined below. All of the experimental protocols were approved by the Northeast Agricultural University Animal Care and Use Committee prior to the initiation of the study.

### Laboratory evaluation

Serum alanine aminotransferase (ALT), aspartate aminotransferase (AST), alkaline phosphatase (ALP), triglycerides (TG), glucose (GLU), total protein (TP), albumin (ALB), high-density lipoprotein (HDL), low-density lipoprotein (LDL) and total cholesterol (TC) were determined using a Beckman CX4 automatic biochemical analyzer (Beckman Coulter, Inc., USA).

### Staining of tissue sections for pathology

Fresh liver tissue pieces from both groups of mice were fixed in 10% neutral formalin, embedded in paraffin, sliced and stained with H&E. The slides were examined by a pathologist to detect the presence of fat, necrosis, fibrosis and inflammation. The pathologist scored them from 0 to 4 using the standards proposed by Dixon for assessing changes in fat and inflammation [35]. The sum of the fat, necrosis, inflammation and fibrosis scores was termed the total histological score. The presence of NAFLD in mice was diagnosed using standard criteria [36].

### Extraction and derivatization of hepatic fatty acid

Frozen liver tissue samples (100 mg) were homogenized in 1 mL of anhydrous diethyl ether. The homogenates were subsequently extracted three times with anhydrous diethyl ether and vigorous vortexing for 1 min, as previously described [37]. After centrifugation, the organic layers were collected and combined, and the solvent was evaporated at room temperature under a nitrogen stream. The dry residue was redissolved in 1 mL of 0.5 mol/L KOH-methanol in a sealed vial in a 60°C bath under an nitrogen stream for 10 min. Next, 1.5 mL of 13% methanolic BF_3 _was added, and the mixture was incubated at 60°C for 30 min. Fatty acid methyl esters were extracted with 1 mL of hexane and 2 mL of saturated sodium chloride and separated and dried under nitrogen stream. The dry residue was redissolved in 25 μL of hexane and prepared for gas chromatography mass spectrometry (GC-MS) by sealing the hexane extracts under nitrogen.

### Liver fatty acid analysis

The derivatives were analyzed by GC-MS (HP6890N-5973 Agilent, Hewlett-Packard, USA) with an electron energy of 70 eV and a source temperature of 230°C. The target compound (fatty acid methyl ester) was detected by full-scan monitoring-mode recording of the fragment ions at an m/z of 30 to 550. Calibration curves were constructed over a concentration range of 0.25 to 125 μg/mL for unsaturated compounds and 0.1 to 100 μg/mL for saturated compounds. Heptadecanoic acid (C17:0) was used as an internal standard. The calculations were based on the assumption of identical detector responses for both the unsaturated and saturated fatty acid. Calibration curves were generated for each of the individual fatty acid and used for liver fatty acid quantification. They were based on the peak-area ratios and linear in their tested ranges.

### Data analysis

A statistics package (SPSS, version 13.0) was used for the statistical analyses. The subject characteristics were expressed as the mean ± standard deviation (SD). The means of each of the fatty acid were compared using unpaired Student's t-tests. Statistical significance was considered to be P < 0.05.

## List of abbreviations used

ALB: albumin; ALP: alkaline phosphatase; ALT: alanine aminotransferase; AST: aspartate aminotransferase; GC-MS: gas chromatography mass spectrometer; GLU: glucose; HDL: high-density lipoprotein; LDL: lower-density lipoprotein; MUFA: monounsaturated fatty acids; NAFLD: nonalcoholic fatty liver disease; PUFA: polyunsaturated fatty acids; SFA: saturated fatty acids; TC: total cholesterol; TFA: total fatty acids; TG: triglyeride; TP: total protein; UFA: unsaturated fatty acids.

## Competing interests

The authors declare that they have no competing interests.

## Authors' contributions

XW and Pro. XZ designed the experiments and contributed to the description and writing. GG contributed to feed mice and did animal treatments, and contributed to the tissue collected. YC contributed to the fatty acids extraction and assay, YF contributed to the data analysis. All authors read and approved the final manuscript.

## Authors' information

Dr. Xin Wang, associate Professor in College of Animal Science and Veterinary Medicine, Heilongjiang Bayi Agricultural University. He is a master supervisor, be major in pharmacology and Chinese herb medicine research.

## References

[B1] Musso G Gambino R Cassader M Recent insights into hepatic lipid metabolism in non-alcoholic fatty liver disease(NAFLD) Prog Lipid Res 2009 48 1 26 10.1016/j.plipres.2008.08.001 18824034

[B2] Sanyal AJ Campbell-Sargent C Mirshahi F Nonalcoholic steatohepatitis: association of insulin resistance and mitochondrial abnormalities Gastroenterology 2001 120 1183 1192 10.1053/gast.2001.23256 11266382

[B3] Chitturi S Abeygunasekera S Farrell GC NASH and insulin resistance: insulin hypersecretion and specific association with the insulin resistance syndrome Hepatology 2002 35 373 379 10.1053/jhep.2002.30692 11826411

[B4] Pinto HC Baptista A Camilo ME Valente A Sargoca A Moura MC Nonalcoholic steatohepatitis. Clinicopathological comparison with alcoholic hepatitis in ambulatory and hospitalized patients Digest Dis Sci 1996 41 172 179 10.1007/BF02208601 8565753

[B5] Jansen PL Non-alcoholic steatohepatitis Eur J Gastroen Hepat 2004 16 1079 1085 10.1097/00042737-200411000-00001 15489564

[B6] Donnelly KL Smith CI Schwarzenberg SJ Jessurun J Boldt MD Parks EJ Sources of fatty acids stored in liver and secreted via lipoproteins in patients with nonalcoholic fatty liver disease J Clin Invest 2005 115 1343 1351 10.1172/JCI23621 PMC1087172 15864352

[B7] Goldberg IJ Ginsberg HN Ins and outs modulating hepatic triglyceride and development of nonalcoholic fatty liver disease Gastroenterology 2006 130 1343 1346 10.1053/j.gastro.2006.02.040 16618425

[B8] Videla LA Rodrigo R Araya J Poniachik J Oxidative stress and depletion of hepatic long-chain polyunsaturated fatty acids may contribute to nonalcoholicfatty liver disease Free Radic Biol Med 2004 37 1499 1507 10.1016/j.freeradbiomed.2004.06.033 15454290

[B9] Araya J Rodrigo R Videla LA Increase in long-chain polyunsaturated fatty acid n-6/n-3 ratio in relation to hepatic steatosis in patients with non-alcoholic fatty liver disease Clin Sci 2004 106 635 643 10.1042/CS20030326 14720121

[B10] De la Maza MP Hirsch S Nieto S Petermann M Bunout D Fatty acid composition of liver total lipids in alcoholic patients with and without liver damage Alcohol Clin Exp Res 1996 20 1418 1422 10.1111/j.1530-0277.1996.tb01143.x 8947319

[B11] Mavrelis PG Ammon HV Gleysteen JJ Komorowski RA Charaf UK Hepatic free fatty acids in alcoholic liver disease and morbid obesity Hepatology 1983 3 226 231 10.1002/hep.1840030215 6832713

[B12] De Almeida IT Cortez-Pinto H Fidalgo G Rodrigues D Camilo ME Plasma total and free fatty acids composition in human non-alcoholic steatohepatitis Clin Nutr 2002 21 219 223 10.1054/clnu.2001.0529 12127930

[B13] Puri P Baillie RA Wiest MM A lipidomic analysis of nonalcoholic fatty liver disease Hepatology 2007 46 1081 1090 10.1002/hep.21763 17654743

[B14] Pan SY Yang R Dong H Yub ZL Ko KM Bifendate treatment attenuates hepatic steatosis in cholesterol/bile salt-and high- fat diet-induced hypercholesterolemia in mice Eur J Pharmacol 2006 552 170 175 10.1016/j.ejphar.2006.09.011 17046746

[B15] Das SK Balakrishnan V Mukherjee S Vasudevan DM Evaluation of blood oxidative stress-related parameters in alcoholic liver disease and nonalcoholic fatty liver disease Scand J Clin Lab Inv 2008 68 323 334 10.1080/00365510701673383 18609067

[B16] Anstee QM Goldin RD Mouse models in non-alcoholic fatty liver disease and steatohepatitis research Int J Exp Pathol 2006 87 1 16 10.1111/j.0959-9673.2006.00465.x PMC2517349 16436109

[B17] Riediger ND Othman R Fitz E Pierce GN Suh M Moghadasian MH Low n-6: n-3 fatty acid ratio, with fish- or flaxseed oil, in a high fat diet improves plasma lipids and beneficially alters tissue fatty acid composition in mice Eur J Nutr 2008 47 153 160 10.1007/s00394-008-0709-8 18454337

[B18] Farrell GC Larter CZ Nonalcoholic fatty liver disease: from steatosis to cirrhosis Hepatology 2006 43 S99 112 10.1002/hep.20973 16447287

[B19] Weber LWD Boll M Stampl A Hepatotoxicity and mechanism of haloalkane: carbon tetrachloride as a toxicological model Crit Rev Toxicol 2003 33 105 136 10.1080/713611034 12708612

[B20] Fraenkel E Lazurova I Feher J Role of lipid peroxidation in non-alcoholic steatoheaptitis Orv Hetil 2004 145 611 618 15119115

[B21] Kohjima M Enjoji M Higuchi N Re-evaluation of fatty acid metabolism- related gene expression in nonalcoholic fatty liver disease Int J Mol Med 2007 20 351 358 17671740

[B22] Barreyro FJ Kobayashi S Bronk SF Werneburg NW Malhi H Gores GJ Transcriptional regulation of Bim by FoxO3A mediates hepatocyte lipoapoptosis J Biol Chem 2007 282 27141 27154 10.1074/jbc.M704391200 17626006

[B23] Brenner RR Nutritional and hormonal factors influencing desaturation of essential fatty acids Prog Lipid Res 1981 20 41 47 10.1016/0163-7827(81)90012-6 7342101

[B24] Cook HW The influence of trans-acids on desaturation and elongation of fatty acids in developing brain Lipids 1981 16 920 926 10.1007/BF02534998 7329212

[B25] Fontana L Moreira E Torres MI Periago JL Sanchez de Medina F Gil A Effects of dietary polyunsaturated fatty acids and nucleotides on tissue fatty acid profiles of rat with carbon techachloride-induced liver damage Clin Nutr 1999 18 93 101 10.1016/s0261-5614(99)80058-2 10459066

[B26] Nebert DW Karp CL Endogenous functions of aryl hydrocarbon receptor(AHR): intersection of cytochrome P450-1(CYP1)-metabolized eisocanoids and AHR biology J Biol Chem 2008 283 36061 36065 10.1074/jbc.R800053200 PMC2606007 18713746

[B27] Capanni M Calella F Biagini MR Prolonged n-3 polyunsaturated fatty acid supplementation ameliorates hepatic steatosis in patients with non-alcohoic fatty liver disease: a pilot study Aliment Pharm Therap 2006 23 1143 1151 10.1111/j.1365-2036.2006.02885.x 16611275

[B28] Das UN Essential fatty acids: Biochemistry, Physiological and Pathology Biotechnol J 2006 1 420 439 10.1002/biot.200600012 16892270

[B29] Levy JR Clore JN Stevens W Dietary n-3 polyunsaturated fatty acid decrease hepatic triglycerides in fischer 344 rats Hepatology 2004 39 608 616 10.1002/hep.20093 14999679

[B30] Di Marzo V Arachidonic acid and eicosanoids as targets and effectors in second messenger interactions Prostag Leukotr Ess 1995 53 239 254 10.1016/0952-3278(95)90123-x 8577777

[B31] Schievella AR Regier MK Smith WL Lin LL Calcium-mediated translocation of cytosolic phospholipase A2 to the nuclear envelope and endoplasmic reticulum J Biol Chem 1995 270 30749 30754 10.1074/jbc.270.51.30749 8530515

[B32] Simopoulos AP Evolutionary aspects of diet, the omega-6/omega-3 ratio and genetic variation: nutritional implications for chronic diseases Biomed Pharmacother 2006 60 502 507 10.1016/j.biopha.2006.07.080 17045449

[B33] Fréneaux E Labbe G Letteron P Inhibition of the mitochondrial oxidation of fatty acids by tetracycline in mice and in man:possible role in microvesicular steatosis induced by this antibiotics Hepatology 1988 8 1056 1062 10.1002/hep.1840080513 3417225

[B34] Labbe G Fromenty B Freneaux E Effects of various tetracycline derivatives on in vitro and in vivo beta-oxidation of fatty acids, egress of triglycerides from the liver, accumulation of hepatic triglycerides and mortality in mice Biochem Pharmacol 1991 41 638 641 10.1016/0006-2952(91)90640-q 1997011

[B35] Dixon JB Bhathal PS Hughes NR O'Brien PE Nonalcoholic fatty liver disease: Improvement in liver histological analysis with weight loss Hepatology 2004 39 1647 1654 10.1002/hep.20251 15185306

[B36] Kleiner DE Brunt EM Natta MV Design and validation of a histological scoring system for nonalcoholic fatty liver disease Hepatology 2005 41 1313 1321 10.1002/hep.20701 15915461

[B37] Foleh J Lees M Sloame Stanley GH A simple method for the isolation and purification of total lipides from animal tissues J Biol Chem 1957 226 497 509 13428781

